# Can We Predict the Evolution of Depressive Symptoms, Adjustment, and Perceived Social Support of Pregnant Women from Their Personality Characteristics? a Technology-Supported Longitudinal Study

**DOI:** 10.3390/ijerph17103439

**Published:** 2020-05-14

**Authors:** Laura Andreu-Pejó, Verónica Martínez-Borba, Carlos Suso-Ribera, Jorge Osma

**Affiliations:** 1 Nursing Department, Universitat Jaume I de Castelló, Castelló de la Plana, 12071 Valencia, Spain; pejo@uji.es (L.A.-P.); borba@uji.es (V.M.-B.); susor@uji.es (C.S.-R.); 2 Instituto de Investigación Sanitaria de Aragón, 50009 Zaragoza, Spain; 3 Departmento de Psicología y Sociología, Universidad de Zaragoza, 44003 Teruel, Spain

**Keywords:** information and communication technologies, pregnancy, personality, depression, adjustment, social support

## Abstract

*Background*: Research exploring the relationship between personality and important pregnancy outcomes (i.e., depressive symptoms, adjustment, and perceived social support) tends to be cross-sectional, arguably due to the difficulties of conducting longitudinal and mental health research in this population. The objective of this study is to use a web-based solution to longitudinally explore how personality traits are associated, not only with the co-occurrence of these outcomes but also with their evolution during pregnancy. Stability and change of these outcomes will also be investigated. *Methods*: The sample included 85 pregnant women attending several medical centers in Spain. The web-based assessment included sociodemographic and obstetric variables (ad hoc) and personality (at the second trimester only), and outcomes at both the second and the third trimester (i.e., depressive symptoms, adjustment, and perceived social support). *Results*: The results showed that adjustment worsened from the second to the third trimester of pregnancy. Neuroticism (N), low extraversion (E), and psychoticism (P) were cross-sectionally and longitudinally associated with outcomes. In addition, N and, to a lesser extent P, uniquely contributed to the evolution of these outcomes in the multivariate analyses, including autoregressions. *Conclusion*: Personality and especially N and P should be evaluated early during pregnancy mental health screening. The use of a web page appears to be a useful tool for that purpose. Technologies might also help disseminate mental health prevention programs for these women, which would be especially recommended for those with a personality profile characterized by high N and P and, to a lesser extent, low E.

## 1. Introduction

Pregnancy is a period of great changes and demands for women and often challenges their ability to adapt to important physiological, social, and psychological changes and to continue to perform despite these difficulties [1]. Additionally, there is a large body of research suggesting that pregnant women may be particularly vulnerable to the detrimental effects of environmental stressors on their mental health [2], which altogether would explain the existence of high emotional distress and stress in this period of the woman’s life [3]. For example, it is estimated that prenatal depression (PD) can affect up to 25%–38% of women worldwide [4]. In Spain, studies on the prevalence of PD are scarce, although the available data indicates that at least 14.8% of women would experience moderate-to-severe symptoms of depression during pregnancy [5]. In addition, in Spain, the healthcare services provided to the perinatal women are mainly focused on the physical aspects related to pregnancy and postpartum, living aside the mental health aspects [6]. Thus, the study of mental well-being during the perinatal period and its predictors is relevant in this country if routine practices are to be changed. The clinical practice guidelines developed by the most prominent organizations in the field of perinatal mental health agree in emphasizing the importance of developing screening strategies that facilitate the detection of women who present risk factors for emotional problems throughout the perinatal period [7,8,9,10]. The risk factors for PD that are most frequently included in these clinical guidelines are current anxious and depressive symptoms, previous history of psychiatric problems, history of sexual abuse or child maltreatment, history of gender violence, adjustment problems with the partner, the experience of a traumatic birth, and the death of the baby during childbirth. In addition to these factors, there is evidence to suggest that certain normal personality traits, especially high neuroticism (N), high psychoticism (P), and low extraversion (E), are related to greater psychopathology in this population [11,12,13]. However, these guidelines do not yet support the need for the evaluation of these personality traits in pregnant women, which suggests that further research is needed in this field.

Regarding N, this is a personality trait characterized by the tendency to experience frequent and intense negative emotions in response to a stressful situation (e.g., pregnancy or childbirth). Furthermore, N is associated with a perception of ineffective coping. Thus, when N is high and persistent, processes such as worry, rumination, or emotional avoidance are likely to appear [14]. Interest in this personality trait, which is considered a widespread biological vulnerability factor for the etiology and maintenance of emotional disorders, including depression, has increased in recent years [15]. For example, Bunevicius et al. [16] found that high scores in N were, together with an unplanned and unwanted pregnancy, independent determinants of prenatal depressive disorders throughout pregnancy. In this same line, several authors have concluded that, of all personality dimensions, N could be considered the most important predictive risk factors for depression both in pregnancy and in the postpartum [13,17]. Additionally and linked with this tendency to experience negative emotions, N has been associated with low perceived social support [18] and poor adjustment to childbirth stressors [19].

Different to N, E refers to the tendency to experience positive emotions such as happiness, optimism, or enthusiasm and attitudes of security, activation, and interest for social interaction [20,21] and is associated with reduced vulnerability to affective disorders [22], increased social support [18], and successful adjustment to childbirth stressors [19]. In this sense, recent studies argue that low E, also known as introversion, would be a key personality trait associated with the onset of emotional disorders and poor adjustment during the perinatal period [23]. 

Finally, P is a personality trait that includes severe psychopathological conditions, such as deception or interpersonal alienation, to more frequent human expressions such as hostility, anger, and social isolation [24]. The literature exploring the role of P in pregnant women is scarce, but research so far supports the idea that high P poses a risk for perinatal women of PD [25]. The relationship between P and adjustment and social support during pregnancy remains unexplored. However, because P is inversely related to agreeableness [26], a key factor associated with perceived availability of social support [18] and effective coping use and adaptation during pregnancy [27], a negative impact of P on these outcomes would be expected.  

To date, the relationship between personality and outcomes in perinatal research (i.e., depressive symptomatology and adaptation to the challenges associated with the perinatal period) has been predominantly explored using cross-sectional designs [13,16,17,25]. Consequently, little is actually known about the influence of normal personality traits (N, E, and P) in the evolution of depressive symptoms and adjustment of women during the perinatal period. Additionally, while it has been argued that poor social support, which is known to negatively impact mood and adjustment during the perinatal period [28,29], might be partly influenced by the personality profile of the mothers (i.e., high N, low E, and high P would arguably represent the high-risk profile) [13,17,20], this also remains unexplored during pregnancy. Note that social support and adjustment are key factors associated with well-being in the mother. Social support (i.e., perceived support from family, partners, and peers) is important both during pregnancy and at the postpartum, and low perceived support appears to add to the mental burden associated with the perinatal period (i.e., depression) [29]. Regarding maladjustment, this measure of poor emotional adaptation to the challenges that can occur during the perinatal period has been associated not only with suffering in the mother but also with internalizing problems in the baby [30]. In the light of the previous, the goal of the present investigation is to investigate whether the personality profile of prenatal women indeed predicts the evolution of depressive symptoms, adjustment to pregnancy, and social support perceived by these women using a longitudinal design.

As noted in the previous lines, there is an evident lack of longitudinal and prospective studies that try to clarify the role of personality traits and other psychosocial variables with respect to important perinatal outcomes, such as depressive symptoms and adjustment [31]. It is possible that this lack of longitudinal research is due to its high cost, especially in terms of time, because longitudinal research implies carrying out repeated evaluations during pregnancy. This might indeed problematic for public health systems since the time available for consultations is limited and estimated (i.e., 10–15 min per patient globally) [32]. Furthermore, reluctance to face-to-face psychological evaluations by women in the perinatal stage are frequent as a consequence of the stigma associated with mental illness [33]. 

The use of information and communication technologies (ICTs) might help overcome some of these limitations of traditional paper-and-pencil, face-to-face evaluations in the perinatal period. Briefly stated, repeated assessment using ICTs imposes less burden on healthcare professional and users, facilitates data collection since evaluations are carried out in the real context of women and traveling to the clinic for assessments is no longer needed, and minimizes stigmatization as they are perceived as being more anonymous and private [34]. Additionally, it is important to note that the use of ICTs in the general population has grown considerably in the past years. In 2008, 61% of homes in the European Union had access to the Internet. Now, in 2019, this has risen to 87% of homes [35]. Most importantly, in our field, 57% of pregnant women download health-related apps to seek information associated with pregnancy, which suggests that the use of ICTs in this field is growing significantly [36]. For this reason, the use of ICT in the field of mental health problems and maladjustment prevention [37], and specifically in the screening of depressive symptoms [38], is becoming increasingly popular and more feasible than ever. Particularly in Spain, where the public health system is very frequently used by the population, the implementation of such ICT solutions would be particularly useful to reduce the current burden associated with face-to-face assessments and interventions [36].

However, despite these promising benefits of ICT use in perinatal settings, most of the screening studies are cross-sectional and do not include the evaluation of risk factors for mental distress and poor adjustment to pregnancy [39]. From this need arises (Mamáfeliz (MMF; HappyMom)), a project that studies the risk factors for the development of perinatal emotional disorders longitudinally through the Internet (i.e., a web page). Thus, the main objective of this study is to explore how certain personality traits are associated, not only with the co-occurrence of depressive symptoms, maladjustment, and poor social support but also with their evolution during pregnancy. Importantly, a web application will be used for the longitudinal assessment of study variables. In addition to exploring the predictive role of personality on outcomes, we will also investigate the stability and change of depressive symptoms, adjustment, and social support during pregnancy. Stability and change will be investigated both at the order level (what is called “differential continuity”) and at the group mean level (what is called “mean-level change”). We hypothesize scoring high in N and P will present a deterioration of depressive symptoms, adjustment, and perceived social support. By contrast, we anticipate that women scoring high in E will report an improvement in outcomes (i.e., reduction in depression and maladjustment and increase in social support). With respect to changes in outcomes, these will be investigated in an exploratory manner due to the limited literature in this regard. 

## 2. Materials and Methods 

### 2.1. Participants

The study sample consisted of 85 pregnant women who voluntarily agreed to participate in the project MMF and to be evaluated with a website throughout pregnancy. Women responded to two evaluations during pregnancy (completers), that is, between weeks 16–24 (Time 1) and weeks 30–36 of gestation (Time 2).

The mean age of the participants was 33.54 years (SD = 4.06; range 25–42). Of these, 94.1% were Spanish, 81.2% had a partner with whom they lived in the home, 94.1% had higher education, and 67.1% were working at the time of the first assessment. The sociodemographic characteristics of the participants are shown in Table 1.

### 2.2. Method

The study dissemination was made by the midwives of the collaborating centers. Study participation was offered to all pregnant women treated at the collaborating centers, which belong to the public or the private health network of MMF. The eligibility criteria were being a pregnant woman over 18 years of age, being fluent in Spanish, and having Internet access.

The collaborating healthcare personnel delivered the study information in writing along with a unique code for each participant to register into the program MMF. Thus, the entire study was conducted online. Once the participants accessed the application with their code, they had to read and accept the data protection and confidentiality documents and accept the informed consent form.

When the registration was completed, and the sociodemographic, obstetric, and medical data were filled, the evaluation of the main study variables began. Finally, the participants received a thank-you message, and they were informed that they would receive an email to complete the following evaluation in the following trimester. 

The approval of the Ethics and Clinical Research Committees of all the collaborating centers was obtained. 

### 2.3. Instruments

Sociodemographic, obstetric, and personality variables were assessed at Time 1 only (during the second trimester). Study outcomes (depressive symptoms, maladjustment, and social support) were evaluated longitudinally twice (during the second trimester and in the third trimester, to explore changes). Personality was only evaluated once to reduce the burden of assessment, because personality characteristics are relatively stable dispositions [40], and because the study focus was not on evaluating changes in personality but on predicting changes in outcomes based on baseline personality profiles.

Assessment of sociodemographic and obstetric variables (ad hoc items): Participants answered questions about their marital status, educational level, employment status, and economic level. They also responded to questions of an obstetric nature, such as the period of gestation, the history of abortion, pregnancy planning, and the level of pregnancy risk.

*Revised Eysenck Personality Questionnaire* (EPQ-RS) [41,42]: The EPQ-RS was administered to assess the three dimensions of normal personality from the Big Three’s Eysenck model, namely neuroticism, extraversion, and psychoticism. This short version consists of 12 items per dimension in which every item evaluates a series of usual behaviors or ways of thinking or feeling. The questionnaire response format is “Yes” =1 or “No” = 0, where higher scores represent a greater presence of the trait that it evaluates. Regarding their psychometric properties of the EPQ-RS, the three scales have an acceptable internal consistency in women, that is, a Cronbach’s alpha of 0.82 for N, 0.79 for E, and 0.67 for P [42]. Similar results were found in our sample for N (α = 0.82), E (α = 0.77), and P (α = 0.60).

*Edinburgh Postnatal Depression Scale* (EPDS) [43,44]: The EPDS consists of 10 items that evaluate the depressive symptoms experienced during the last seven days. Each item has four response options with a unique value that varies from 0 to 3. Items 1 and 2 are scored from 0 = “As much as always” to 3 = “Not at all”, and items 3–10 are valued inversely, from 3 = “Yes, most of the time” to 0 = “No, never”. The total score is obtained by summing the scores of all 10 items. Higher scores should be interpreted as indicating more severe depressive symptoms. The maximum total score is 30 points. The Spanish adaptation of the scale has obtained very good internal consistency estimates in pregnant women (0.81 ≤ α ≤ 0.85, according to trimester during pregnancy) [43]. In our sample, good internal consistency was found both in the second (α = 0.86) and the third (α = 0.85) trimester of pregnancy.

*Maladjustment Scale* (MS) [45]. This scale consists of 5 items that evaluate to what extent the psychological distress experienced by the person affects different areas of daily life, that is work or studies, social activities, free time, family life, and relationship with their partner. In addition, the scale global item that refers to the overall degree of maladjustment. Each item is rated on a 6-point scale (0 = “None” to 5 = “Very Serious”). Higher scores represent higher maladjustment. Cronbach’s alpha coefficient of the Spanish validation of the scale was 0.94 [45]. In our sample, good internal consistency was found both in the second (α = 0.85) and the third (α = 0.82) trimester of pregnancy.

*Multidimensional scale of perceived social support* (MSPSS) [46,47]. The MSPSS consists of 12 items evaluated on a 7-point Likert-type scale (1 = “Strongly disagree” to 7 = “Strongly agree”). The 12 items were designed to measure the perception of support in three areas: family (items 3, 4, 8, and 11), friends (items 6, 7, 9, and 12) and other significant persons (items 1, 2, 5, and 10). High scores indicate a greater perception of support received in each of the areas. In our study, we selected the family and friends scales to reduce the number of statistical analyses and to minimize the risk of multicollinearity problems. The use of the total score of the scale was discarded due to the interest in differentiating the support of the family from that of other agents, such as friends. The alpha coefficients of the family and friends scales were 0.89 and 0.92, respectively [47]. In our sample, good internal consistency was found both in the second (α = 0.94) and the third (α = 0.94) trimester in the family-scale. Similar results were found in the friends-scale both in the second (α = 0.97) and the third (α = 0.95) trimester.

### 2.4. Data Analysis

First, a descriptive analysis was made. Next, the change in the study variables was explored. Two different procedures were used to investigate the evolution of the study outcomes (i.e., depressive symptoms, adjustment, and social support) during pregnancy. On the one hand, the Student *t*-test for related samples was used to assess the change in mean scores (mean-level change). Additionally, Pearson’s correlations were calculated to explore order changes between the two measurement times (differential continuity).

In order to explore the cross-sectional and prospective associations between baseline personality and outcomes, we first conducted a series of Pearson’s correlations. 

Finally, multivariate regression was carried out to explore to what extent the changes in study outcomes (i.e., depressive symptoms, adjustment, and perceived social support) at Time 2 (third trimester of pregnancy) were explained by personality factors, after controlling for important covariates (i.e., age the corresponding outcome at Time 1, that is during the second trimester of pregnancy). Age was included as a covariate because it has been related to psychosocial adjustment during the perinatal period in the literature [48]. A Holm–Bonferroni correction was used for all statistical analyses, which resulted in a *p*-value of 0.0125.

## 3. Results

### 3.1. Retention of the Participants Into the Online Assessment Program MMF 

**Figure 1:**
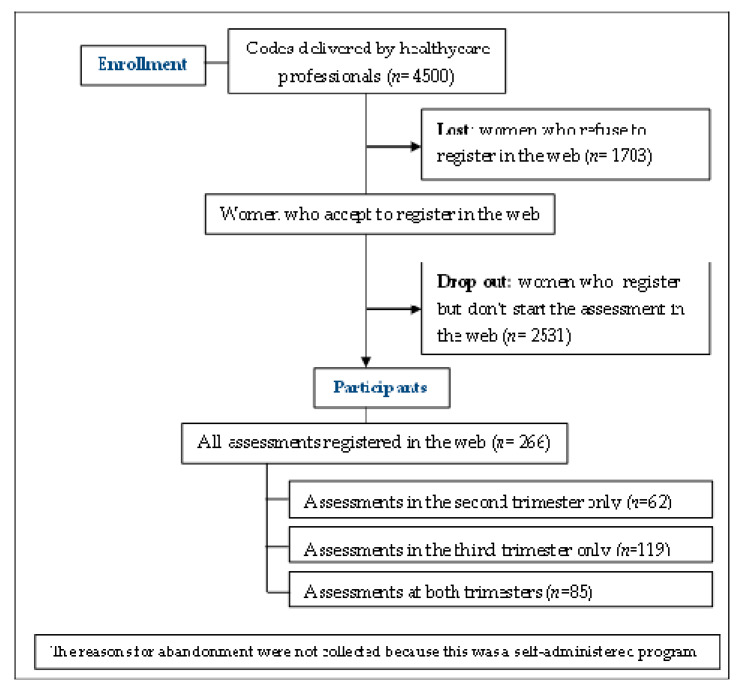
Flow chart of the participants in the study.

Regarding the participation of women in the online program MMF, 4500 women received codes to enroll in the program. Of these, 62.2% (n = 2797) effectively registered into the web, but only 5.9% (n = 266) participated at some point in the prenatal evaluation of the program. Finally, only 85 women completed both evaluations during the second and the third trimester during pregnancy. Figure 1 describes the flow diagram of study participation.

### 3.2. Descriptive Results of the Study Variables

Table 2 shows the means and standard deviations of the women’s scores in the study variables, as well as a comparison with population normative scores. Overall, the women in our study showed a personality profile characterized by low N (*t* = 7.17, *p* < 0.001), average E (*t* = 1.50, *p* = 0.135), and low P (*t* = 6.04, *p* < 0.001). The levels of depressive symptoms during pregnancy in our sample were comparable to those of normative populations (all *p* > 0.0125). Perceived social support by family (*t* = 3.59, *p* < 0.001) and friends (*t* = 4.55, *p* < 0.001) was higher in our sample. Maladjustment scores could not be compared because normative scores do not exist for women.

### 3.3. Evolution of Outcomes (Depressive Symptoms, Adjustment, and Perceived Social Support) During Pregnancy 

Table 3 presents the means and standard deviations of study outcomes, as well as the analyses of change at the mean level and at the rank order level. Regarding mean values, only a significant increase in maladjustment was observed during pregnancy (*t* = 4.83, *p* < 0.001). Taking changes at the order level, the moderate-to-strong significant correlations should be interpreted as indicating some stability at the order level. Social support variables were the ones that remained more stable in terms of order (strong correlation). 

### 3.4. Bivariate Associations Between Personality Variables, Depressive Symptoms, Adjustment, and Perceived Social Support

Table 4 shows the bivariate associations between the main study variables. The results of the correlations between the variables evaluated at Time 1 (second trimester) showed that N was associated with greater depressive symptoms (*r* = 0.61, *p* < 0.001), greater maladjustment (*r* = 0.29, *p* = 0.007), and poorer perceived family (*r* = −0.31, *p* = 0.004) and friends support (*r* = −0.34, *p* = 0.002). Conversely, high levels in E were associated with a decreased depressive symptoms (*r* = −0.43, *p* < 0.001), reduced maladjustment (*r* = −0.30, *p* = 0.006), and increased perceived support by the family (*r* = 0.34, *p* < 0.001) and friends (*r* = 0.34, *p* = 0.002).

The cross-sectional associations between study variables evaluated in Time 2 (third trimester) showed family support (*r* = −0.38, *p* < 0.001) and friends support (*r* = −0.47, *p* < 0.001) were associated with decreased depressive symptoms. Support from friends (*r* = −0.34, *p* = 0.001) and depressive symptoms (*r* = 0.44, *p* < 0.001) were inversely associated with maladjustment.

Finally, the results of the longitudinal correlations showed that N at Time 1 was prospectively related to more severe depressive symptomatology (*r* = 0.54, *p* < 0.001), maladjustment (*r* = 0.46, *p* < 0.001), and poorer support from family (*r* = −0.29, *p* = 0.008) and friends (*r* = −0.48, *p* <0.001) at Time 2. Similarly, P at Time 1 was related to greater depressive symptomatology (*r* = 0.33, p = 0.002) and less support from family members (*r* = −0.29, *p* = 0.007) and friends (*r* = −0.34, *p* = 0.002) at Time 2. Conversely, E at Time 1 was associated with less intense depressive symptoms (*r* = −0.29, *p* = 0.008) and maladjustment (*r* = −0.36, *p* < 0.001), as well as with increased support from the family (*r* = 0.28, *p* = 0.010) and friends (*r* = 0.38, *p* < 0.001) at Time 2.

Different to personality, age was not cross-sectionally or longitudinally related to outcomes (all *p* > 0.050).

### 3.5. Personality Factors as Predictors of Depressive Symptoms, Maladjustment, and Perceived Social Support Evolution During Pregnancy: Multivariate Analysis)

Table 5 presents the results of the multivariate regression analyzes. In order to decide whether potential covariates should be included in the regressions, we investigated the bivariate associations between economic level, abortion history, pregnancy risk, and history of childbirth. None of the correlations were significant and they were all very small in size (all *r* < 0.20, all *p* > 0.05), which might have been influenced by the low heterogeneity of responses (approximately 75% of women had moderately high economic levels, did not have previous abortions, and had very a low risk pregnancy and over 65% of them were primiparous). Thus, there was no evidence to support their inclusion in the multivariate regression and doing so would only increase the risk of multicollinearity.

Across personality variables, both N (*β* = 0.40, *t* = 2.62, *p* < 0.001, 95% CI = 0.10, 0.71) and P (*β* = 0.58, *t* = 2.62, *p* < 0.001, 95% IC = 0.10, 0.71) uniquely and significantly contributed to the prediction of depressive symptoms at a prospective level after controlling for the effect of baseline depressive symptoms. In relation to maladjustment, the only personality factor to prospectively and significantly contribute to this outcome above and beyond baseline maladjustment levels was N (*β* = 0.50, *t* = 2.99, *p* < 0.0125, 95% CI = 0.17, 0.84). Finally, and similar to depressive symptomatology, N (*β* = −0.07, *t* = −2.65, *p* < 0.0125, 95% CI = −0.12, −0.02) and P (*β* = −0.10, *t* = −2.58, *p* < 0.0125, 95% CI = −0.18, 0.02) were the personality dimensions to significantly and uniquely contribute to the prediction of perceived social support from friends at a prospective level and after controlling for baseline perceived friends support. Regarding perceived family support, the results did not indicate that personality variables contributed to the evolution of this outcome.

## 4. Discussion

The present study aims at providing further evidence into the role of personality (N, E, and P) and important pregnancy outcomes, namely, depressive symptoms, adjustment to pregnancy challenges, and perceived social support. In order to achieve this goal and in order to address some limitations observed in the literature, this investigation included two major improvements compared to previous studies, namely, the implementation of a longitudinal design and the use of ICTs for assessment. Overall, the results indicated an important contribution of N and, to a lesser extent, P on the evolution of study outcomes, but failed to reveal a significant prospective association between E and outcomes when controlling for the remaining personality factors and baseline outcome scores. Additionally, while the inclusion of ICTs for technology made the longitudinal evaluation more feasible for healthcare professionals, response rates by participants were very poor, which will be discussed in more detail in the following lines. 

Indeed, one of the strengths of the present study was the longitudinal investigation of the evolution of important perinatal outcomes. In this sense, the results showed that significant mean-level changes only occurred in maladjustment, so that women reported a poorer adaptation when comparing the second and the third trimester of pregnancy. As evidenced in past research, pregnancy imposes major changes in the lifestyles of women and requires important psychological resources for an adequate adaptation to these changes [1], which means that pregnancy can become a stressful period for women [49]. An interesting finding was that, according to our results, it is at the last stage during pregnancy when women experience greater limitations in their life areas (e.g., work activity, leisure, or social life). Based on our data, these increased difficulties in adaptation did not result in a proportional depressed mood, which suggests that a deterioration of adjustment with time might have been expected by the participants or was well tolerated at an emotional level. While acknowledging the aforementioned increase in adjustment challenges in the third trimester due to biological reasons, it is also true that preventive interventions focused on offering information about changes during pregnancy and guidelines to better adjust to such changes, such as maintaining social and leisure activities and physical activity, could help mitigate the degree of maladjustment in this period of pregnancy. As stated in previous research, ICTs can help to provide information-based interventions, physical exercise, or behavioral activation in perinatal women at reduced costs and high disseminability [37,50,51].

As noted in the previous lines, depressive symptomatology did not change during pregnancy in the present study, which contrasts with some past research [16]. These differences could be explained by several reasons, such as the sample sizes used in previous investigations, the country of origin of women participating in the studies, or their educational level, which was very high in the present study and this is known to be a protective factor for depression [52]. In addition, it is also possible that our participants have present protective factors for depression, such as an adequate evolution of pregnancy, an overall good health status, or adequate coping strategies. 

Similar to depression, no changes were observed in perceived social support, which was high at both assessment times. This is an important finding considering that social support seems to have a buffering effect on women’s health during the perinatal stage and, as some authors affirm, social support could contribute to reducing the impact of stress and depressive symptomatology [48]. Related to this, the fact that in the present study baseline support from family and friends was prospectively associated with reduced depressive symptomatology is consistent with the idea that social support facilitates the journey to motherhood [53]. 

Regarding the relationships between personality and outcomes, our results showed significant associations both at the cross-sectional and longitudinal level between personality traits and outcomes. Consistent with the literature, N and E showed the strongest associations with outcomes, and P was mostly associated with the social variables included in the study (i.e., social support) [16,17]. As expected, women who presented high N and low E scores reported more depressive symptoms, poorer adjustment, and less social support cross-sectionally. This adds to the existing literature showing that N is a risk factor for well-being and adaptation to challenges, such as those experienced during pregnancy, while E would be a protective factor in such situations [11,17,25]. 

Interestingly and one of the key contributions of the present study was the finding that the aforementioned contribution of personality, particularly in the case of N and P, was preserved in the multivariate analyses, including prospective data and controlling for the auto-regressions. This design allowed us to confirm that personality is not only associated with prenatal outcomes in a cross-sectional manner, but that N and P might be risk factors for the deterioration of many of these outcomes. It is important to note that personality explained an important percentage of the variance of prospective outcomes (13.3% for depressive symptoms, 11.2% for adjustment, and 10% for perceived social support), considering baseline scores in the outcomes were controlled for. While cross-sectional associations are indeed important and informative, the prospective relationships evidenced in the present investigation add to past research in suggesting that, if prevention or intervention programs are not provided to populations at risk (i.e., high N and P), they are likely to continue to worsen in the mental symptomatology, adaptation, and social satisfaction during pregnancy, which puts them at risk for their own well-being and that of the newborns [54]. 

Note that one of the present study goals was to investigate whether personality characteristics were indeed associated with social support during pregnancy. Our results support past research in showing that E is associated with satisfaction with social life, and N and P are inversely associated with this outcome [40]. Because social support was inversely associated with depressive symptoms and maladjustment, these results support the idea that intervention programs should be addressed to women at risk for poor perceived social support (i.e., those with a low E and high N and P profile). Again, ICT solutions might be useful tools to provide social support to women during pregnancy [55].

Contrary to our expectations, age did not show significant associations with any of the study variables. The literature is ambiguous when establishing a relationship between age and the risk of depression during pregnancy [11]. While some studies suggest that younger women are at greater risk of developing depressive symptoms [56], inconsistent findings have also been reported [57]. These discrepancies are probably due to differences in the mean age of women from the different studies. However, a review by Biaggi et al. [11] indicated no association between age and perinatal outcomes, which is consistent with our findings and suggests that older age might not be a key protective factor for well-being and adjustment in pregnant women. 

A final contribution of the present study was the implementation of a web-based application for data collection. From our experience, the use of a web page for the longitudinal evaluation of risk factors and associated psychological variables during pregnancy has represented a simple, inexpensive, and confidential alternative that we believe helped overcome some of the current barriers for the longitudinal assessment of mental health problems in perinatal care, most notably the insufficient consultation time [34,58]. Additionally, research suggests that the application of ICT facilitates perinatal mental health screening due to the reduced stigma associated with non-face-to-face evaluations, the flexibility with which assessments can be completed (anywhere and anytime), and the reduced costs in terms of professional time [50,59].

While acknowledging the benefits of ICT for mental health screening, as noted by our low participation and retention rates, the implementation of online longitudinal assessments with very limited professional involvement is still challenging. One of the main barriers for participation, as informally reported by some of the participants, was associated with the time required to complete the evaluation protocol, which was perceived as being too extensive. As indicated by recent research conducting longitudinal and ecological momentary assessments, a reduction of items (e.g., validation of individual item measures against full traditional scales) and the selection of key questionnaires only is fundamental in design with technology and longitudinal designs [60]. The use of apps, which are more accessible and acceptable for perinatal women, might help as well in this direction [39]. Finally, the inclusion of gamification or treatment elements (i.e., tips to be used during pregnancy) might also facilitate engagement with longitudinal studies [61].

### Limitations

This study has some limitations that should be taken into account. First, note that the sample is composed of a group of women with very similar sociodemographic characteristics, which limits the generalization of our findings. The small sample size (N = 85) also represents a problem. In this sense, it is important to consider the main obstacle associated with using the Internet in self-applied screening, prevention, or treatment programs, which is low adherence. In our case, as noted in the previous lines, the fact that this was a longitudinal study with a significant load in terms of the number of questionnaires to be completed might have negatively impacted on sample participation and retention. Note, however, that this is a frequent finding in the perinatal research using technology, even when treatment is provided [62]. As noted earlier, there are a number of initiatives that could be implemented to minimize this problem when conducting longitudinal assessments. Another limitation refers to the reliance on self-report measures only, which might have led to interpretation bias or social desirability. However, and in relation to social desirability, the anonymous and online nature of the evaluations should have encouraged honesty [63]. 

## 5. Conclusions

In sum, our results support the idea that certain personality characteristics (high N and P) might pose a risk to women for a deterioration of important well-being outcomes. This is important for prevention purposes and would suggest that women characterized by high N or high P should be monitored more frequently during pregnancy (e.g., using ICTs). Additionally, in order to promote changes in personality tendencies, self-applied programs, again using ICTs, could be the first solution in a stepped manner of care, which is more feasible than offering face-to-face individual treatments for all, considering the limited existing resources in public health settings in Spain [64].

It is important to note that an intervention program specifically aimed at reducing N and the negative consequences derived from this vulnerability factor currently exists [14]. This intervention, called the Unified Protocol, allows individuals to acquire a set of emotional regulation strategies that facilitate tolerance to discomfort derived from intense emotions, such as sadness or anxiety [65]. Future studies should test if the Unified Protocol effectively reduces N levels in a sample of pregnant women and whether, consequently, an improvement in outcomes (e.g., depressive symptoms, adaptation to pregnancy, and perceived social support) occurs. Again, and according to previous similar research, ICTs could be used to conduct and easily disseminate these interventions in women in the perinatal stage [60]. This opens fascinating avenues for future research in the field.

## Figures and Tables

**Table 1:** Sociodemographic characteristics of the sample (N = 85).

	Total
Nationality	
Spanish	94.1%
Latin American	2.4%
Western Europe	3.5%
Marital Status	
With a living partner	81.2%
Without a living partner	18.8%
Educational level	
≤12 years of education	5.9%
>12 years of education	94.1%
Employment situation	
Working	67.1%
Unemployment	20%
Sick leave	12.9%

**Table 2:** Descriptive statistics of study variables and comparison with normative scores.

Variables	N	Pregnant Women M (*SD*)	N	Reference Female Population M (*SD*)	*t*	*p*	*d*
Personality	85		583				
EPQ-RS (N)		3.8 *(3.1)*		6.6 *(3.4)*	7.169	<0.001	0.86
EPQ-RS (E)		8.7 *(2.7)*		8.2 *(2.9)*	1.498	0.135	0.18
EPQ-RS (P)		2.1 *(1.8)*		3.8 *(2.5)*	6.043	<0.001	0.78
Depressive symptoms	85		569				
EPDS (2nd T)		5.2 *(4.6)*		5.7 *(3.9)*	1.076	0.282	0.12
EPDS (3rd T)		4.7 *(4.3)*		5.7 *(4.3)*	1.999	0.046	0.23
Maladjustment	85						
MS		6.2 *(5.3)*		-	-	-	-
Social support	85		265				
MSPSS (Family)		6.5 *(0.8)*		6.0 *(1.2)*	3.592	<0.001	0.49
MSPSS (Friends)		6.3 *(1.0)*		5.6 *(1.3)*	4.549	<0.001	0.60

EPQ-RS = Eysenck Personality Questionnaire (N: neuroticism; E: extraversion; P: psychoticism); EPDS = Edinburgh Postnatal Depression Scale; T = trimester; MS = maladjustment scale; MSPSS = Multidimensional Scale of Perceived Social Support.

**Table 3:** Evolution of study outcomes from the second (Time 1) to the third trimester (Time 2).

	Time 1		Time 2		*t*	95% CI	*r*
	M	*SD*	M	*SD*			
**EPDS**	5.2	4.6	4.7	4.3	−1.024	−1.42,0.46	0.53 **
**MS**	6.2	5.3	8.9	5.4	4.832 **	1.59, 3.80	0.54 **
**MSPSS (F)**	6.5	0.8	6.4	1.0	−0.744	−0.15, 0.07	0.85 **
**MSPSS (Fr)**	6.3	1.0	6.1	1.0	−1.493	−0.29, 0.04	0.71 **

M = mean; SD = standard deviation; *t* = Student’s *t* ; *r* = Pearson’s correlation; EPDS = Edinburgh Postnatal Depression Scale; T = trimester; MS = maladjustment scale; MSPSS = Multidimensional Scale of Perceived Social Support; F = family; Fr = friends. * *p* (Holm Bonferroni sequential correction) < 0.0125; ** *p* < 0.001.

**Table 4:** Correlations between personality, age, and outcomes (i.e., depressive symptoms, adjustment, and social support).

	2	3	4	5	6	7	8	9	10	11	12
***Second trimester***											
**1. Neuroticism**	−0.36 **	0.19	−0.31 *	−0.34 *	0.61 **	0.29 *	0.10	−0.29 *	−0.48 **	0.54 **	0.46 **
**2. Extraversion**		−0.09	0.34 **	0.34 *	−0.43 **	−0.30 *	−0.12	0.28 *	0.38 **	−0.29 *	−0.36 **
**3. Psychoticism**			−0.34 *	−0.19	0.11	0.03	−0.09	−0.29 *	−0.34 *	0.33 *	0.08
**4. Family support**				0.65 **	−0.23	−0.20	−0.05	0.85 **	0.67 **	−0.33 *	−0.29 *
**5. Friends support**					−0.27	−0.18	0.04	0.60 **	0.71 **	−0.31 *	−0.16
**6. Depression**						0.67 **	0.13	−0.18	−0.28 *	0.53 **	0.48 **
**7. Maladjustment**							0.11	−0.13	−0.22	0.27	0.54 **
**8. Age (third trimester)**								−0.10	−0.04	0.18	0.15
**9. Family support**									0.71 **	−0.38 **	−0.27
**10. Friends support**										−0.47 **	−0.34 *
**11. Depression**											0.44 **
**12. Maladjustment**											

In bold, the intercorrelations between variables at the second trimester and their corresponding variable at the third trimester. * *p* (Holm Bonferroni sequential correction) < 0.0125; ** *p* < 0.001.

**Table 5:** Multivariate regression predicting study outcomes.

	DV	Beta	*t*	95% CI	*R*^2^ Change	*F* Change
	**Depression (T2)**					
***Block 1: depression (T1)***		0.28	2.70 **	0.07, 0.50	0 .279	32.15 **
***Block 2: age (T1)***		0.14	1.56	−0.04, 0.33	0.013	1.55
***Block 3: personality (T1)***					0.133	6.08 **
**Neuroticism**		0.40	2.62 **	0.10, 0.71		
**Extraversion**		−0.02	−0.16	−0.33, 0.28		
**Psychoticism**		0.58	2.84 **	0.17, 0.99		
	**Maladjustment (T2)**					
***Block 1: maladjustment (T1)***		0.42	4.44 **	0.23, 0.60	0.291	34.09 **
***Block 2: age (T1)***		0.09	0.74	−0.15, 0.32	0.009	1.09
***Block 3: personality (T1)***					0.112	5.04 *
**Neuroticism**		0.50	2.99 *	0.17, 0.84		
**Extraversion**		−0.26	−1.35	−0.64, 0.12		
**Psychoticism**		0.03	0.12	−0.48, 0.55		
	**Family support (T2)**					
***Block 1: family support (T1)***		0.95	12.48 **	0.80, 1.10	0.719	212.81 **
***Block 2: age (T1)***		−0.02	−1.03	−0.04, 0.01	0.004	1.08
***Block 3: personality (T1)***					0.001	0.06
**Neuroticism**		−0.06	−0.31	−0.05, 0.03		
**Extraversion**		−0.07	−0.31	−0.05, 0.04		
**Psychoticism**		−0.04	−0.12	−0.07, 0.06		
	**Social support friends (T2)**					
***Block 1: friends support (T1)***		0.56	7.39 **	0.41, 0.71	0.506	84.95 **
***Block 2 : age (T1)***		−0.01	−0.68	−0.05, 0.02	0.005	0.82
***Block 3: personality (T1)***					0.100	6.73 **
**Neuroticism**		−0.07	−2.65 *	−0.12, −0.02		
**Extraversion**		0.03	1.15	−0.03, 0.09		
**Psychoticism**		−0.10	−2.58 *	−0.18, 0.02		

T1 = second trimester; T2 = third trimester. * *p* (Holm Bonferroni sequential correction) < 0.0125; ** *p* < 0.001.
